# Flanking Variation Influences Rates of Stutter in Simple Repeats

**DOI:** 10.3390/genes8110329

**Published:** 2017-11-17

**Authors:** August E. Woerner, Jonathan L. King, Bruce Budowle

**Affiliations:** 1 Center for Human Identification, University of North Texas Health Science Center, 3500 Camp Bowie Blvd., Fort Worth, TX 76107, USA; jonathan.king@unthsc.edu; 2 Center of Excellence in Genomic Medicine (CEGMR), King Abdulaziz University, Jeddah 22252 3270, Saudi Arabia

**Keywords:** stutter, flanking variation, longest uninterrupted stretch (LUS), parental allele length (PAL), short tandem repeats (STR)

## Abstract

It has been posited that the longest uninterrupted stretch (LUS) of tandem repeats, as defined by the number of exactly matching repeating motif units, is a better predictor of rates of stutter than the parental allele length (PAL). While there are cases where this hypothesis is likely correct, such as the 9.3 allele in the TH01 locus, there can be situations where it may not apply as well. For example, the PAL may capture flanking indel variations while remaining insensitive to polymorphisms in the repeat, and these haplotypic changes may impact the stutter rate. To address this, rates of stutter were contrasted against the LUS as well as the PAL on different flanking haplotypic backgrounds. This study shows that rates of stutter can vary substantially depending on the flanking haplotype, and while there are cases where the LUS is a better predictor of stutter than the PAL, examples to the contrary are apparent in commonly assayed forensic markers. Further, flanking variation that is 7 bp from the repeat region can impact rates of stutter. These findings suggest that non-proximal effects, such as DNA secondary structure, may be impacting the rates of stutter in common forensic short tandem repeat markers.

## 1. Introduction

Stutter, an artifact commonly occurring during the amplification of short tandem repeats (STRs), has important ramifications for mixture interpretation. The amount of signal at a stutter position and at other allelic positions of a profile is considered when determining the potential of a minor contributor(s)’s alleles being part of the stutter signal. Thus, understanding the rate and variance of stutter is an essential part of mixture interpretation (for minor and trace contributions). Likewise, mis-modeling of these rates and variances may either reduce power, in the case that variances are artificially elevated to encompass unmodeled parameters, or they may induce bias, if some of these covariates are known and left unmodeled. The most frequent stutter type is a −1 repeat. There are two highly correlated definitions of a −1 repeat: (1) by allele length, wherein an amplicon with a fragment size corresponding to one less repeat unit of an allele is observed, and (2) by nucleotide state, wherein an amplicon with one less repeating subunit (by sequence) of an allele is observed. The latter definition is implicitly tied to a particular subunit. For example, in the case of a compound STR with two repeating subunits (or motifs) there are likewise two −1 stutter states each with (possibly) different rates; one rate for the first repeating subunit’s state, another rate for the second repeating subunit’s state, and perhaps some measure of the covariance between these rates. Assessing the rates of stutter in simple repeat markers, where by definition there is only one repeating subunit, mitigates the difference between these two measures perhaps to negligible levels.

A major predictor of the rate of stutter is the longest uninterrupted stretch (LUS) of tandem repeats, wherein the increasing length of LUS alleles is directly related to increasing rates of stutter [1,2,3,4,5]. It has been posited that the LUS is a better predictor of rates of stutter than the parental allele length (PAL) [1,4,6] (but see [7]). These observations are based on particular loci, or even particular haplotypes of a given locus.

**Figure 1:**
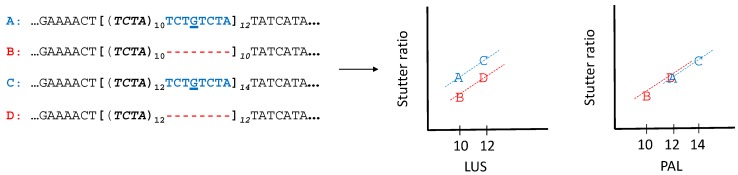
A cartoon of four different haplotypes in a shared slopes model. Four haplotypes shown have a longest uninterrupted stretch (LUS) (bold, parentheses) of either 10 (A, B), or 12 (C, D). A mutation (G) has induced a quasi-repeat that is inside of the canonical repeat region (brackets), but is not itself repeating. This quasi-repeat is adjacent to the repeating motif (and thus is a type of flanking variation) which causes the LUS (parentheses) to differ from the parental allele length (PAL) (in this case, demarcated by the brackets). If the quasi-repeat behaves as an actual repeat (e.g., haplotype A behaving not like a 10, but a 12), then the stutter ratio of the A allele will be similar to that of the D allele. This can be seen if one evaluates stutter as a function of the LUS (left scatterplot) or as a function of the PAL (right scatterplot). A shared slopes linear regression model fits a line with a different intercept but the same slope for the red versus blue haplotypes (see Appendix A). Note that quasi-repeats are but one mechanism by which the LUS and the PAL can differ in simple repeat regions.

A more complex interpretation proposes that different allelic backgrounds may be associated with different rates of stutter (Figure 1). Different allelic backgrounds, residing in the flanking haplotypes, may have arisen because of insertion/deletion (indel) polymorphisms in, near, and/or perhaps adjacent to the repeat region. Likewise, single nucleotide polymorphisms (SNPs) may have occurred on the periphery of a repeat region, which have the potential to create quasi-repeats that may extend the LUS effect (e.g., the effect of rs9546005 and rs202043589 on the stutter of D13S317). In such cases, the LUS and the PAL may differ, and which is a better predictor of stutter has yet to be formally assessed. In other words, while the LUS has a straightforward definition, its definition is based on exactly identical repeats in a string (restricted to the canonical repeat region). This strict definition may not always be biologically appropriate. For example, a CATA [GATA]_10_ allele could behave as an allele with 10 repeats or as an allele with 11 repeats. While this distinction may not be meaningful in the case where the initial C is fixed in the population, it may become meaningful if the C is polymorphic. Because of this variation in the region adjacent to the repeat, there are at least two haplotypic backgrounds on which STR variation can arise. Thus, the two types of 10 repeats (by LUS) can be compared, depending on the haplotypic background. While this example placed the flanking variant adjacent to the repeat, nonadjacent variants also create differential haplotypic backgrounds, and these variations may influence rates of stutter.

This study explores rates of stutter as predicted by both the LUS and the PAL on differing haplotypic backgrounds in a large sampling of alleles taken from multiple populations. As there are numerous confounding variables in such an assessment, this analysis is performed solely in the simplest situation, i.e., simple repeats, wherein there is only one repeating unit. Different flanking haplotypes within loci were identified and characterized, and the types and locations of the variants involved were described. The rates of stutter were then contrasted both across loci and across haplotypic backgrounds within loci to assess whether flanking sequence variation, as well as the LUS, influence the rates of stutter. Linear models were then constructed so that the significance and the size of the effects observed could be described. These models, and the visualizations of these models, were then assessed to determine if the LUS is a better predictor of stutter than the PAL, or if these effects are locus-specific and too varied to draw a generalized conclusion.

## 2. Materials and Methods

### 2.1. Samples, Sequencing, and Haplotype Identification

The samples used in this study are a subset of the individuals sequenced in [8]. Briefly, 714 unrelated individuals from four population groups (US Caucasian, *N* = 200; Hispanic, *N* = 189; African American, *N* = 167; and Chinese; *N* = 158) were sequenced with the ForenSeq^TM^ Signature Prep Kit using the MiSeq FGx Forensic Genomics System both from Illumina (San Diego, CA, USA). Sequence variation was characterized with STRait Razor v2s [9]. STRait Razor was used to capture both the STR and flanking variation, including noise variation, as a single haplotype per allele at each simple repeat locus across individuals. 

Each individual’s true haplotype(s) was identified using the STRait Razor v2s Analysis Suite, allowing for separation of noise haplotypes, including stutter, from true haplotypes. While it is possible to identify a particular haplotype as “noise”, there can be ambiguity as to the origin of that haplotype (e.g., a length 9 repeat in the presence of a 10/11 heterozygote could arise from a −1 stutter event from the 10 or a −2 stutter event from the 11, see [10]). To mitigate the effects of this uncertainty, three classes of genotypes were considered: (1) if the locus was homozygous, defined at the level of the haplotype, then both alleles were used; (2) otherwise, the difference in the length-based allele call was computed. If this difference was ≤1, then neither allele was used; and if this difference was <3, then only the shorter allele was used; and (3) otherwise both alleles and their −1 stutter products were considered in the analysis.

### 2.2. Repeat and Flanking Region Characterization

Haplotypes, as defined by their character strings, were reduced into their 5′ flanking sequences and 3′ flanking sequences, and their repeat structure as follows: The canonical repeat region (as defined in [11]) was identified using the Excel-based analysis tools found in STRait Razor v2s [9]. STRait Razor then was used to characterize the repeat region, reducing the region into its more succinct repeat region sequence summary (e.g., reducing a hypothetical haplotype in the repeat region of D2S441 TCTATCTATCTATCTGTCTA into [TCTA]_3_ TCTG TCTA) as per the forensic definitions of [11].

While the repeat regions defined in [11] are essential for the forensic characterization of STRs, their definition requires some amendment for the goal of this study to characterize −1 stutter in simple repeats, and the location of the simple repeat need not be fixed in a population. For example, the locus D2S441 has two types of 10 alleles (as defined by length), one that is [TCTA]_10_ and another that is [TCTA]_8_ TCTG TCTA. Thus, one could suggest that the repeat region is itself variable, spanning 10 repeat-units in the former haplotype and 8 units in the latter haplotype. It remains an open question as to whether stutter models should treat both alleles as a 10, or one as a 10 and the other as an 8, or if these alleles have fundamentally different stutter profiles. Of further note, while in this example the effective repeat region would be reduced in some samples, the presence of particular SNPs in the region adjacent to the repeat can similarly cause the effective repeat region to expand and span different nucleotides in some individuals.

To accommodate both of these effects, all repeat region sequence summaries were reprocessed, considering the first and the last motif (as the canonical repeat region defined in [11]) with respect to their corresponding flanks. If additional adjacent matching motifs in the flanking sequences were found, those motifs were added to the repeat sequence summary and removed from the corresponding flanking region for that haplotype. After this processing, any leading or trailing sequence in the repeat region that was not itself repeated (considering any number >1 as repeating) was then added to the corresponding flanking region (e.g., the TCTG TCTA in the above example would not be considered as a repeat, despite being in the canonically defined repeat region).

After the repeat itself was characterized, each unique flanking haplotype was also characterized. In particular, the flanking 5′ and 3′ regions were concatenated, and each unique concatenation was given a unique index (an integer), allowing for the association between the repeat, regardless of the repeating motifs in the repeat region, and the flanking region. The flanking indexes were then placed in descending order by frequency, such that index 1 corresponds to the most common flanking haplotype, and index 2 corresponds to the second-most common flanking haplotype, etc.

### 2.3. Identifying Simple Repeats and Their −1 Stutter Products

Simple repeats consist of a single repeating motif unit and perhaps contain microvariants that can interrupt this motif. The analysis herein takes a rigid definition of simple repeats. In particular, after the above processing, if a single repeating motif remained, then the haplotype was considered to contain a simple repeat. As the above examples imply, some length-based alleles are implicitly removed in this process (e.g., the 9.3 allele of the TH01 locus), while some loci are removed entirely (Table 1).

While stutter can be assessed by the sequence of the alleles or relative to the length of the parental allele, this study focuses on the former as follows: The numbers of correct reads (*C*) and −1 stutter products (*S*_−1_) were tabulated. *C* was taken as the number of records that correspond to the correct haplotype (i.e., measured by reads), and *S*_−1_ (also measured by reads) was taken as the number of exactly matching sequences with exactly one less repeat as defined by nucleotide sequence. The LUS was then computed considering the state of the nucleotides; the PAL was computed using the length of the allele, and likewise, the stutter ratio (SR) was defined as:

(1) $$\frac{S_{-1}}{C}$$

### 2.4. Modelling of Stutter Ratios

The SR was modeled using robust linear regression with the lmrob function in the robustbase package in the statistical package R (version 3.4.2) [12]. Briefly, robust linear regression differs from ordinary least squares (OLS) linear regression in several ways (see [13]). In particular, several assumptions are made by OLS approaches that are likely not to hold for the modeling performed in this study. The robust regression implemented in the lmrob library addresses some of these issues (in particular, the issues of heteroscedasticity and sensitivity to outliers) by performing an iterated reweighted least squares linear regression, wherein outliers are identified based on their residuals, down-weighted, and the coefficients are re-inferred in an iterative procedure. Tests for heteroscedasticity were performed using bptest function in the lmtest package in R. Coefficient estimates and outlier-resilient and heteroscedasticity-consistent confidence intervals were estimated using the robust regression techniques implemented in the lmrob function in R set to the default parameters [13]. Both OLS regression and robust regression are sensitive to multicollinearity (i.e., from correlation amongst the independent variables). To address this, variance inflation factors (VIFs) were computed. VIFs measure how inflated each covariate’s coefficient is due to multicollinearity, with VIFs >10 generally being considered high. VIFs were computed using vif function in the car library in R. Separate regressions were run on each locus, and nested regression models were compared using a Wald test as implemented in the ANOVA function in R. Note that modeling of the effects of the flanking sequence was only performed in the cases in which there were at least two flanking haplotypes with an *N* ≥ 25. Bivariate local regressions (i.e., loess), contrasting *S*_−1_ to *C*, were performed using the default smoothing parameters as found in the ggplot2 graphical library (version 2.2.1) in R [14].

## 3. Results

### 3.1. The Distribution of Flanking Haplotypes

**Figure 2:**
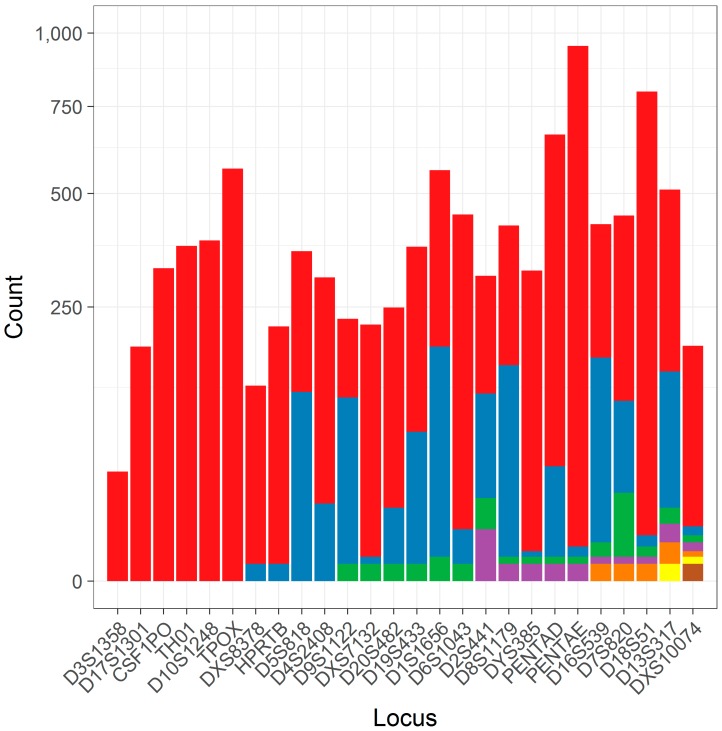
A column plot of the number of alleles found for different flanking haplotypes within loci. Each column corresponds to a different locus, which is contrasted against the number of alleles for that locus (*y*-axis, note square root scale). The number of alleles constituting the different flanking haplotypes (ordered left to right) is shown in color under an arbitrary color scheme.

After data preprocessing (see Methods and Materials), a total of 9995 separate allele calls at 26 loci were characterized as simple repeats that could be utilized in this study. Each locus had between 1 and 7 unique flanking haplotypes (median 3; Table 1), though many of these haplotypes are at low frequency (Figure 2) and may be less amenable to statistical analyses due to issues stemming from limited sampling.

### 3.2. Comparing the Longest Uninterrupted Stretch to the Parental Allele Length

**Figure 3:**
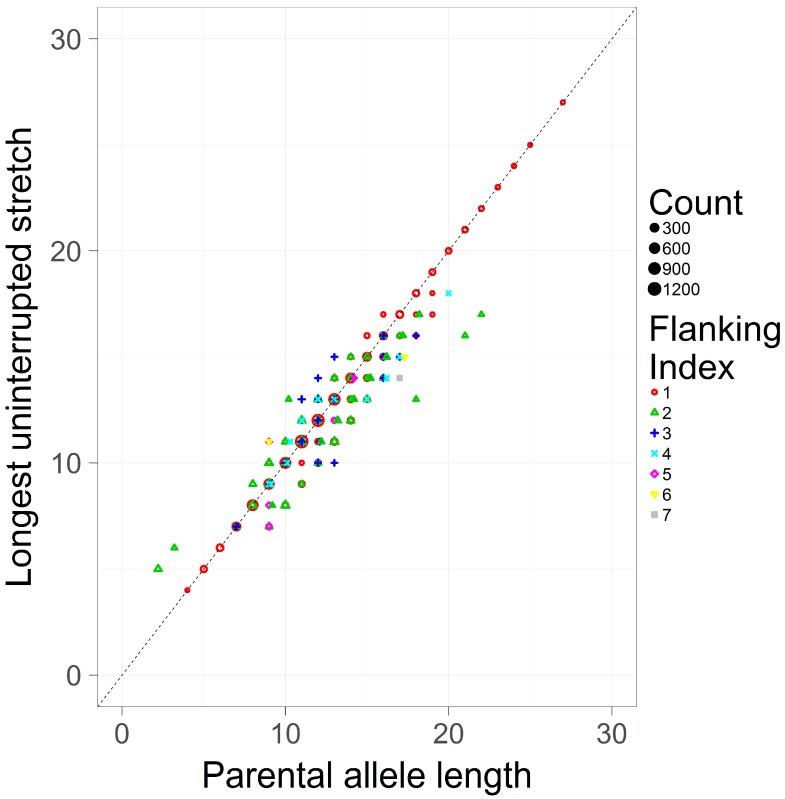
A scatterplot of the LUS and the PAL in a pooled sample of simple repeats. The LUS (*y*-axis), is contrasted against the PAL (*x*-axis). Different flanking indexes (shapes and colors) correspond to unique flanking haplotypes, with lower indexes corresponding to higher frequency haplotypes (also shown by size) from the pooled population dataset of [8].

This study only considers simple repeats, where by definition there is only one hypermutable repeating unit. Given this, the LUS and the PAL should be largely consistent. To assess this assumption, the LUS and the PAL were pooled across loci and contrasted (Figure 3). The Pearson correlation coefficient between these two measures is high (*r* = 0.974). As the PAL is an estimator of the LUS in the case of simple repeats, it is perhaps expected that both definitions are in general agreement, though they are not identical. As seen in Figure 3, the most common flanking haplotype (flanking index 1) tends to correspond to the inferred allele length (i.e., on the main diagonal the LUS and the PAL are equal), however less frequent haplotypes (index >1) are typically found off the diagonal, suggesting that the same length-based allele call may correspond to substantively different repeat counts.

### 3.3. Coverage and the Stutter Ratio

As a ratio statistic, evaluations of the SR may be misleading when considered without evaluating its constitutive components. For example, correlations between *S*_−1_ and *C* may complicate interpretations of the SR (e.g., a ratio of 0.2 might have a continuum of meanings, depending on, say, *C*). If we treat *C* as a proxy of the amount of template DNA, then the mean stutter ratio is likely invariant to *C* (though the same is not true of the variance) [15]. Likewise, any relationship between *C* and SR cannot be attributed to the well-described effects of low-template DNA processing (e.g., increased PCR cycles) [16,17,18], as the PCR/sequencing conditions are held constant. Conversely, such correlations may be possible for reasons both bioinformatic and chemical. With respect to the latter, stutter products may exhibit preferential amplification. Sequence length may also be a factor because sequence quality decreases near the end of the read making the bioinformatic assessment of *C* reads more difficult than that of *S*_−1_ reads. Thus, because of these reasons, *C* alleles may be captured at lower rates than *S*_−1_ alleles, which in turn may induce a correlation between *S*_−1_ and *C*. Correlations aside, the variance in the SR may be considered too high for statistical analyses if there are too few supporting reads (as per [15]).

**Figure 4:**
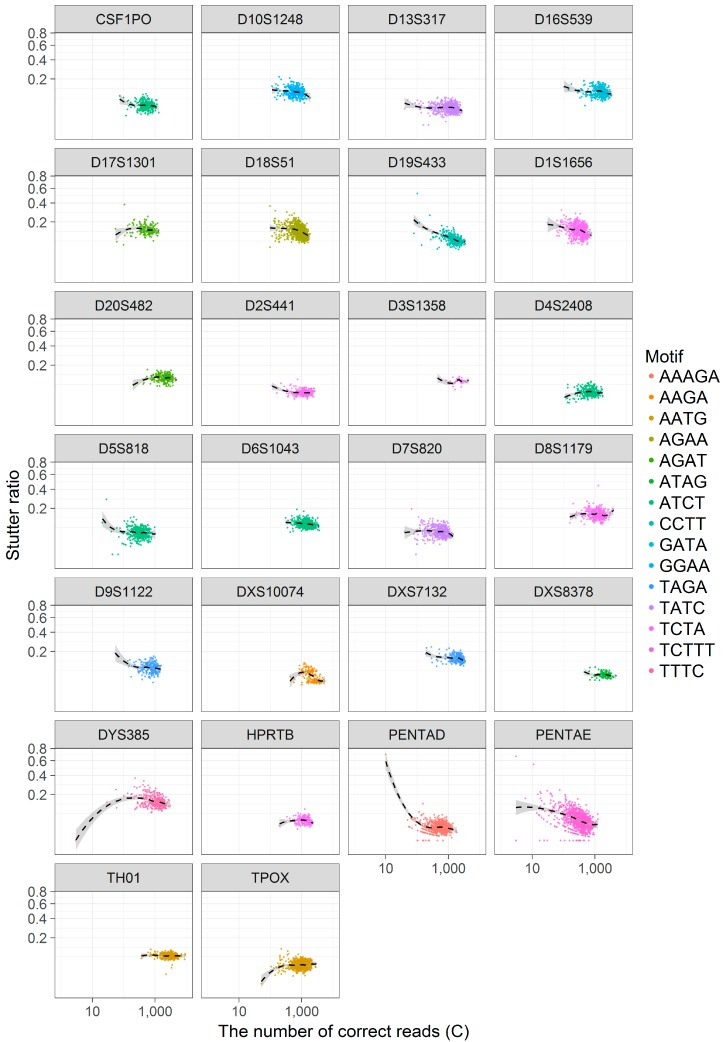
A scatterplot of the stutter ratio (SR) compared to the number of correct reads (*C*). The stutter ratio is defined as $\frac{S_{-1}}{C}$, where *S*_−1_ is the number of −1 stutter reads and *C* is the number of correct reads. The SR (*y*-axis) is contrasted against *C* (*x*-axis) (note square-root and log scaling) across loci (inner plots). Within each inner plot, separate local regressions were fit to show smoothed trend lines (black dashed lines). The repeating motif is shown by color so as to associate similar motifs across loci.

To visually assess these hypotheses, the SR was contrasted against *C* across loci (Figure 4) and loess trendlines with 95% confidence intervals were fitted separately across loci to aid in visual interpretation. As seen in Figure 4, there are at least three apparent trends: *S*_−1_ and *C* appear to be negatively correlated, regardless of *C*, in some loci (e.g., D19S433, PENTA D, PENTA E), while other loci show no such correlations (e.g., TH01, D8S1179, though both loci are at high coverage). Still other loci appear to have a biphasic relationship between *S*_−1_ and *C*, wherein for small values of *C* (~<100) there is an apparent correlation between these values, and for larger *C* this correlation dissipates (e.g., D5S818, D9S1122, D4S2408). This last observation may be driven solely by an increase in the variance in SR when evaluating loci with a small number of reads, though other causal mechanisms cannot be formally excluded. To partially address this issue, haplotype calls with less than 100 supporting (correct) reads were omitted from further analysis.

### 3.4. Characterizing Flanking Sequence Variation

As seen in Figure 2 and Table 1, while some loci surveyed had no flanking variation, others have several flanking haplotypes (see also [8]). In order to characterize how and if the SR depends on flanking sequence variation, a sufficient sample size was sought to explore such hypotheses. Given this constraint, 10 loci were identified that had at least two (and as happens, exactly two) flanking haplotypes each with at least 25 supporting haplotype calls (i.e., both sample sizes ≥25). The two flanking sequences were then manually aligned (i.e., this is equivalent to assuming a constant LUS and aligning the entirety of the captured region) and compared with the alignment placing gaps proximal to the repeat region (Supplementary Table S1). The alignments were then characterized by describing the type of change required to convert the first haplotype (flanking index 1, the ultimate) into the second haplotype (flanking index 2, the penultimate) (Table 2). It should be stressed that the characterization induced by this alignment strategy likely has little evolutionary meaning; its importance is in describing how the flanking haplotypes differ, not in the likely order of events that relate the two haplotypes. For example, the locus D13S317 has two high frequency flanking haplotypes corresponding to rs9546005 A/T in 3′ flank. Index 1 in this study corresponds to the haplotype that has, after the TATC repeat, an additional AATC, while in index 2 that AATC is missing. While there are multiple evolutionary scenarios that can give rise to this particular observation, for the purposes herein this difference was defined as a four-base deletion that is adjacent to the repeat (a definition that naturally arises when the LUS is held constant across the different haplotypic backgrounds, as occurs when these data are placed in a scatterplot or as assessed in the linear models proposed.) Thus, when, for example, two individuals with two different haplotypic backgrounds and the same LUS are compared, the extent to which stutter differs between these two haplotypes can be assessed. Further, by calling this polymorphism a deletion, the direction of the expected effects is given. That is, if the AATC behaves as a TATC, then one might expect that when the LUS is held constant the second haplotypic background has a lower rate of stutter than the first haplotypic background. Similarly, if this hypothesis is placed in a linear regression framework, the hypothesis tested becomes the question of whether the two intercepts for the two flanking haplotypes significantly differ (see also Figure 1).

As seen in Table 2, some loci’s flanking haplotypes (D16S539, D7S820) only differ by a single SNP, others (D19S433) differ by indel polymorphisms that are not adjacent to the repeat, while the remaining have indels that are adjacent to the repeat. Further, amongst the adjacent indel class, some likely arose because of SNPs in the original repeat (e.g., D8S1179, D9S1122), while others necessarily involved an indel polymorphism out of frame of the repeat motif (PENTAD).

### 3.5. Stutter Ratios and Flanking Sequence Variation

The SR is positively correlated with the LUS [3,19], and the extent of this effect varies substantially across loci [1,2,5]. While many important covariates have been identified to explain this heterogeneity across loci, such as GC content, the repeating motif itself [2,20], and whether the repeat is interrupted and/or compound [1,5,21], an alternative approach to control for some of these covariates is to simply allow the coefficients to vary across loci (e.g., [5,22]). Since these variables are either fixed or are highly collinear with the flanking sequence of the locus and/or its LUS, the last approach does not permit one to disentangle the effects of, for example, GC content vs. motif-type in determining the SR. However, these confounding variables are implicitly controlled by evaluating each locus independently.

Longer repeating alleles tend to be associated with higher rates of stutter. However, there is some evidence to support the hypothesis that the LUS (as defined by the nucleotide sequence) may be a better predictor of the SR than the size of the allele [1,4,19]. Viewed more generally, this hypothesis could be reframed to determine if the nucleotides near the repeating segment have a demonstrable effect on rates of stutter. Given this consideration, the SR was contrasted against the LUS and the PAL within each locus. To evaluate the SR as a function of flanking sequence, only loci with at least two flanking haplotypes with an *N* ≥ 25 were considered (Table 2), giving a final sample of 10 loci.

**Figure 5:**
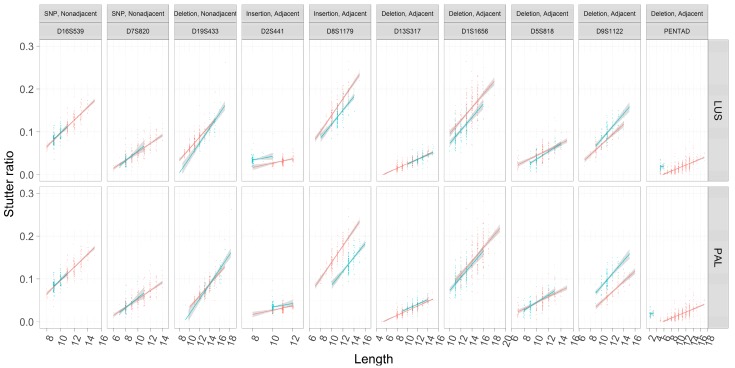
Stutter ratios as a function of the length of the longest uninterrupted stretch (LUS) and the parental allele length (PAL). The stutter ratio (SR) (*y*-axis) was computed across loci (outer plot, columns). The loci chosen have two distinct flanking haplotypes (red and blue points for flanking haplotypes one and two, respectively), and the difference between these haplotypes (given a constant LUS) is described in the outer columns (see also Table 2). A small amount of statistical noise (*x*-axis, called jitter) was added to the length of the LUS (outer plot, top row) and the length of the parental allele (PAL, outer plot, bottom row) to reduce over-plotting. Separate linear regressions with 95% CIs were fit to the corresponding flanking haplotypes within loci. For a formal assessment of slopes and intercepts, and their differences across flanking haplotypes, see Table 3 and Table 4. Each outer column corresponds to the cartoon scatterplots in Figure 1.

These trends were first visualized considering only their marginal relationships (e.g., neglecting the effects of Figure 4) between LUS and SR across different flanking haplotypes within loci (Figure 5). Assessments of the SR were also fit using OLS regression so as to visualize the slopes and intercepts within loci across flanking haplotypes. As has been previously reported, rates of stutter appear to vary among loci [5,22]. Different flanking haplotypes also appear to be associated with different rates of stutter in some cases but not in others (Figure 5).

### 3.6. Linear Models of Stutter Ratios

To formally model the effect seen in Figure 5, robust linear regressions were performed on each locus (Materials and Methods, see also Appendix A). Ten nested pairs of linear models were constructed, one pair for each locus. The first model in the pair is a noninteraction model: *SR ~ LUS + Flank + C*, where *Flank* is an indicator variable set to 1 in the case of the second flanking haplotype (emulated by the blue in Figure 5), and otherwise set to 0 (emulated by the red in Figure 5) (see also Figure 1). Given the trends observed in Figure 5 (e.g., PENTA D), the number of correct reads (C) was also considered in the model which allows for different intercepts (but the same slope) between flanking haplotypes. The second model in the pair is an interaction model: *SR ~ LUS × Flank + C*, which allows for different slopes and intercepts (an explanation of this is given in Appendix A) for the alternate flanking haplotype. As these two models were nested, the model fits of the interaction versus noninteraction models were compared. If the interaction model yielded significantly better model fit than the noninteraction model (i.e., *p* < 0.05), it was chosen as the final model; otherwise the noninteraction model was chosen.

OLS regression makes several assumptions that are likely violated in this application, namely multicollinearity, homoscedasticity, and further the coefficients inferred from OLS regression are sensitive to outliers. To address the last two issues, robust linear regressions were performed, which serve to both down-weight outliers, reducing their impact on the inferred coefficients, and providing heteroscedasticity-consistent coefficient estimates. VIFs were also computed on the non-dummy variables to assess multicollinearity.

Breusch–Pagan tests of heteroscedasticity were performed on the regression models, with the resulting *p-*values being combined assuming independence using the method of Fisher [23]. The combined *p-*values were exceedingly small when considering either the LUS or the PAL (both *p*-values < 10^−36^), suggesting the presence of heteroscedasticity. The VIFs, while sometimes large, were not excessive (generally >10 is considered high, all VIFs <7) (Table 3 and Table 4), suggesting that the correlations between the independent variables are not especially high in this analysis.

The results from the linear regressions are seen in Table 3 and Table 4. All loci, except D5S818, do not display significantly better support for the interaction model than the non-interaction model. The coefficients in Table 3 and Table 4 were modified (added) such that the coefficients shown have both intercept estimates for both flanking haplotypes and the shared slope (or different slope in the case of D5S818) (as per Appendix A). The reported *p*-value reflects the level of statistical support for a difference between the intercepts and slopes between the two flanking haplotypes, as appropriate. Additionally, coefficients for *C* are reported, as is the *p-*value for a significant difference from 0 slope for this variable. All coefficients are unstandardized (typically represented as *b*, as opposed to β), and thus represent changes in the original units modeled (e.g., the coefficient for *C* is in terms of reads), though *C* is considered a nuisance variable in this analysis.

Qualitatively, finding significant support for a difference in intercepts (and not in slope) suggests that both flanking haplotypes likely have the same rate of growth, with respect to the LUS (or PAL, see also Figure 1). The difference in the constant (intercept) suggests they have different starting values (when LUS is 0). Stated more qualitatively, with a difference in intercepts and not slope, a 5 allele on the first haplotype background may behave similar to a 6 allele on the second haplotype background. This distinction is perhaps meaningful if, for example, adjacent to the currently defined repeat region there is some sequence “close” to the repeat (e.g., CCTA adjacent to a TCTA). An open question is whether or not it is generally better to consider the CCTA as part of the repeat. Finding a difference in slopes, however, suggests a different slippage rate on one haplotypic background versus another.

## 4. Discussion

### 4.1. Nonadjacent Flanking Mutations and Stutter

The two loci (D16S539, D7S820) contain flanking haplotypes that differ by a single SNP outside of the canonical repeat region. These loci show consistent trend lines across assessments of the LUS or the PAL (note these are equivalent in this case). No difference is apparent in the trend lines between flanking haplotypes (Figure 5). Further, neither locus shows significant differences with respect to the inferred intercepts, nor do they support models with different slopes (Table 3 and Table 4). These observations suggest that at least in this limited sampling of two loci, SNPs that are nonadjacent to the repeat do not appear to influence rates of stutter.

**Figure 6:**
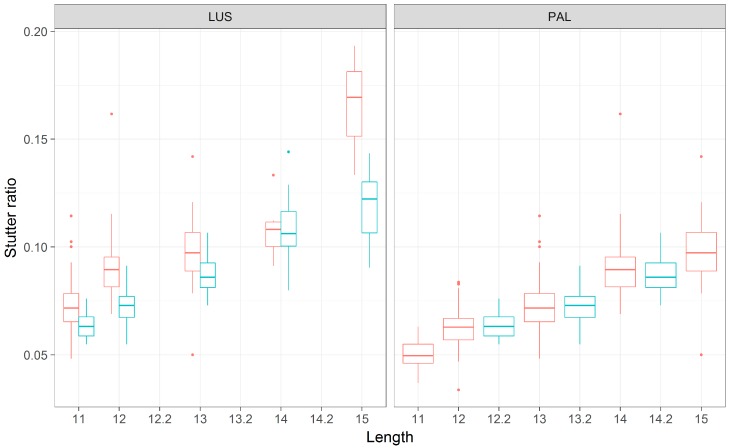
A boxplot of stutter ratios by *LUS* and by *PAL* for the D19S433 locus. Stutter ratios (*y*-axis) were measured as a function of the number of CCTT repeats (LUS, *x*-axis panel 1), and by the PAL (*x*-axis, panel 2) across two haplotypic backgrounds (blue/red). Microvariants with the X.2 allele corresponds to a 2-bp deletion that is not adjacent to the repeating unit for flanking index 2 (blue boxes, flanking index 2). Thus, 12 and 12.2 alleles (PAL) have 10 and 11 CCTT nucleotides (LUS), respectively, yet 12.2 alleles appear to have stutter ratios that are closer to a 12 allele (by LUS), despite their difference in LUS. The *x*-axis is truncated for illustration to only consider LUS in the range [11,15].

The D19S433 locus provides a single example of a nonadjacent indel in the flanking region (i.e., the X.2 alleles described in [24]), and in this case the sequence and length-based characterizations appear to differ (Figure 5). When the TC deletion is incorporated into the length of the repeat (PAL, Figure 5), the trend lines become more similar than if one considers the number of CCTTs (LUS, Figure 5), though note that while the slopes appear to differ in Figure 5, this difference lacks statistical support (Table 3 and Table 4). The regression analyses further refine our comparison between the PAL and the LUS; when assessed by the PAL, the two intercepts are not significantly different (−0.08344 vs. −0.08322, *p* = 0.995), yet when assessed by the LUS, the intercepts are significantly different (−0.0586 vs. −0.0685, *p* < 10^−7^). Finding a difference in the latter and not the former suggests that the intermediate (−0.2) allele length formed by this indel is associated with stutter that is intermediate in magnitude, wherein a 12.2 appears more like a 12 than a 13 (as defined by the PAL) (Figure 6). However, a 12.2 and a 13 allele both have 11 CCTT repeats, suggesting that the LUS is an incomplete predictor of stutter for this locus and the PAL instead should be preferred (as per Figure 1). Thus, it appears that the 2-bp deletion, even though it is ~7 bp away from the closest CCTT repeat, appears to have a stabilizing effect on rates of polymerase slippage at the repeat, reducing the overall rate of stutter by ~1 CCTT repeating unit. Linear models support this supposition, where the difference in intercepts between these haplotypes is 0.010 (Table 4, −0.059–0.069), which is very similar to the effect of an additional CCTT repeat (Table 4, slope coefficient point estimate of 0.012). It should be further noted that the bases involved and/or adjacent to this deletion are similar to the repeating motif itself, which may point to one of the underlying causative mechanisms for this finding.

### 4.2. Adjacent Flanking Mutations and Stutter

The remaining loci’s flanking haplotypes differ by sequence that is characterized as adjacent to the repeat. In these cases, there is no clear correspondence between the trend lines’ relative position and whether or not the flanking haplotypes differ by an insertion or deletion (i.e., blue lines are not always above red lines in the case of insertions, and vice versa). Since insertions and deletions are defined relative to the different flanking haplotypes within a locus (Table 2), the hypotheses that suggest longer alleles must have higher rates of stutter are far too simplistic.

Taking the loci D2S441 and D8S1179 for example, both flanking haplotypes differ by an 8-bp insertion that is 1 SNP away from a 2-repeat unit change (blue versus red lines in Figure 5), and both repeats have the same TCTA repeat motif (Table 2). However, the flanking insertion for the D2S441 locus appears to increase the rate of stutter (blue line on top of red line, Figure 5), while the opposite is true for the D8S1179 locus. Further, the intercept estimates are significantly different when considering the LUS or the PAL (Table 3 and Table 4), suggesting that these eight bases neither behave like two TCTAs (PAL), nor do they behave like a complete lack of two TCTAs (LUS). As these effects are opposite between the D2S441 and D8S1179 loci, by process of elimination this result suggests that other unmodeled variables, or differences in (flanking) GC content or DNA secondary structure may be contributing to these differential rates of stutter.

The D13S317 locus, on the other hand, has flanking haplotypes that differ by an AATC deletion adjacent to the TATC repeat. In this case, only when the PAL is considered, and not the LUS, do the two haplotypes appear to have different intercepts (Figure 5). Despite this observation, the intercepts estimated by the robust regression analysis (Table 3 and Table 4) are significantly different in both cases (intercept coefficients are −0.03134 and −0.02768 for haplotypes 1 and 2, *p* < 1 × 10^−5^ by PAL, and −0.03134 and −0.03331 for haplotypes 1 and 2, *p* = 0.024 by LUS), though note that when considered by LUS the intercepts are more similar. The latter finding may be an example of these differences being statistically, but not biologically, different. Overall, the adjacent TATC at the D13S317 locus largely behaves as unrepeated flanking sequence, which provides evidence that the LUS is likely the more appropriate predictor of stutter in this locus (similar to TH01 [2] and DYS635 [25]).

**Figure 7:**
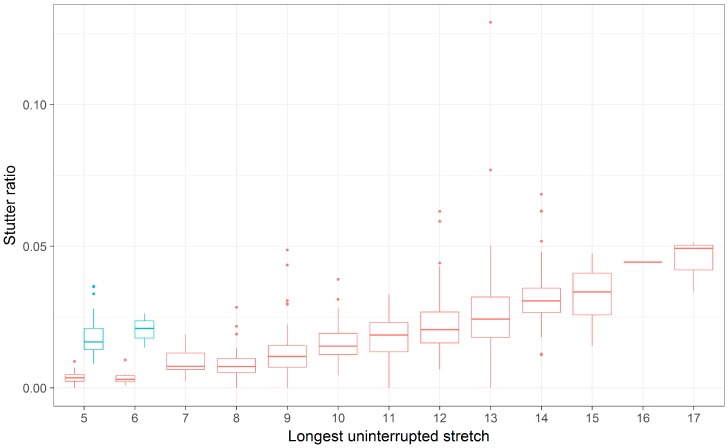
A boxplot of stutter ratios for the PENTA D locus. Stutter ratios (*y*-axis) were measured as a function of the number of repeating AAAGA repeats nucleotides (LUS, *x*-axis). These measurements are contrasted against the two flanking haplotypes (red, blue), wherein the haplotype depicted in blue has a 13-bp deletion adjacent to the repeat region, relative to the haplotype depicted in red. Note that the blue boxes correspond to 2.2 and 3.2 alleles (by PAL).

The PENTA D locus provides yet another example where neither the LUS nor the PAL yield similar intercepts across the two flanking haplotypes (Figure 5 and Figure 7). In particular, the second haplotype background refers to the 2.2 and 3.2 alleles of the PENTA D locus, which differ by a 13-bp deletion adjacent to the repeat (Table 2). When considered by the PAL or the LUS, the SR is elevated over that of individuals without this 13-bp deletion (Figure 5 and Figure 7; Table 3 and Table 4), with the inferred difference in intercepts being significant in both cases. Evaluating the locus by LUS causes the intercepts to be closer than by similar evaluations by the PAL (−0.017 vs. 0.010 by PAL, −0.017 vs. 0.001 by LUS), though these intercepts are still quite dissimilar. This observation suggests that using the number of repeating units is more correct than using the length of the allele (i.e., PAL), though neither approach explains the discrepancy in the rates of stutter between haplotypes in this locus. This result, and in particular the direction of this result, is somewhat puzzling. The deleted sequence (Table 2) bears similarity to the AAAGA motif of these alleles, yet the presence of this similar sequence (haplotype index 1; red in Figure 5 and Figure 7) is associated with greatly reduced rates of stutter. Further, the deleted sequence has 11 As, which are weakly bonded (i.e., two hydrogen bonds), and as per the strand slippage model proposed in [3] should promote strand disassociation and increase slippage [20]. Yet the opposite was observed. Empirically, however, there is support for the hypothesis that weakly bonded nucleotides may be associated with lower rates of stutter [2], though the precise mechanism of this association has yet to be elucidated.

**Figure 8:**
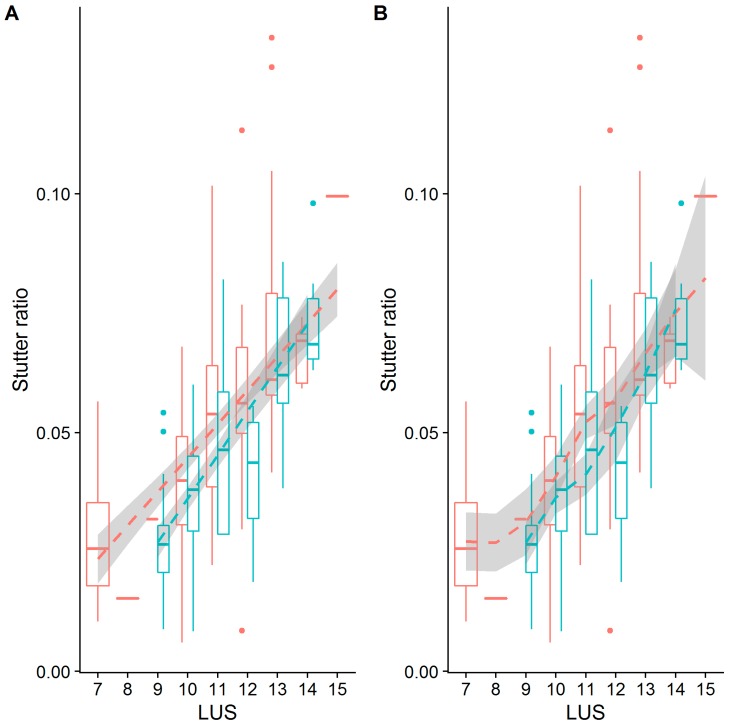
A boxplot of stutter ratios by LUS for the D5S818 locus fit with two types of smoothing. Stutter ratios and the LUS were computed. The trend lines shown are fit from a linear regression (**A**), and using a local regression (loess) (**B**), contrasted against two flanking haplotypes (blue, red).

Only one locus, D5S818, showed evidence for different intercepts and different slopes between the flanking haplotypes (Figure 5, Table 3 and Table 4). While a difference in intercepts is perhaps expected, if, for example, the flanking variant shares sequence similarity with the repeat itself, differences in stutter rates across flanking haplotypes are perhaps unexpected. Given this finding, the D5S818 locus was reassessed considering both a linear model of the stutter ratio and using a local regression (Figure 8). While not a formal analysis, evidence for an apparent difference in slopes can be seen when linear models are considered (panel A in Figure 8). When fit with a local regression (panel B in Figure 8), which allows for more nuanced relationships between these variables, the slopes tend to be more similar. Thus, this difference in the rates of stutter may instead reflect a reduced rate of stutter for seven alleles (by LUS) in this locus, though the reduced rate of stutter for 12 alleles on the second haplotypic background may also contribute to this finding. Thus, it may be that the apparent differences observed in the D5S818 locus with respect to flanking variation may instead be an artifact of modelling.

## 5. Conclusions

This study presents evidence that the rates of stutter vary dramatically, both across loci (as is well established in the literature) and on different haplotypic backgrounds within loci, even in the simplest types of repeats. The findings of this study indicate that there is no clear “best practice” when it comes to modelling the stutter ratio in simple repeats; in some loci, evaluations that use the LUS are more appropriate, and with other loci the PAL should be preferred. These findings also suggest that flanking variation (that is, variation that flanks the repeat itself yet may or may not be in the canonical repeat region) influences rates of stutter, and it further suggests that inclusion of additional independent variables (perhaps in addition to better modeling strategies) are essential to form more correct models of stutter. Reducing the variance in these estimated rates of stutter would be helpful to develop more robust methods of mixture interpretation and probabilistic genotyping. Further, conditioning such inferences on the flanking variation of the primary contributor may reduce these variances and also change the point estimates of the stutter ratio, which likely has marked impacts on downstream inferences on mixtures.

While the evidence so far points to polymorphisms adjacent to the repeat region having impact on the rate of stutter, these rates can also be affected by polymorphisms that are as much as 7 bp away (in this study). Such assessments in more complicated repeats (e.g., complex, compound) may have inherent ambiguities—because of linkage disequilibrium and variation inside of the repeat and outside of the repeat may be linked. Thus, even if a particular mutation is associated with a change in stutter, this association need not be causative. Also, the resulting multicollinearity may make some modeling strategies difficult. Evaluating these rates in simple repeats permits an appreciable sample size of alleles that have the same flanking variation and the same repeat variation, which allows for a relatively easy control on some of these effects. Sufficient sample sizes in more complex repeats are less easily attained, either because they may require exceedingly large sample sizes, or knowledge on how and if different repeat motifs interact to form stutter products (or if these effects can otherwise be neglected).

It should also be stressed that the assessments of stutter in this study were only performed with respect to common haplotypic backgrounds. It follows that less-common variants (e.g., those depicted in Figure 2) may also have subtle and varied impacts on the rates of stutter. Given the complexity of the task of predicting stutter rates even in the simplest of cases, i.e., with simple repeats, accurate stutter modelling of more complex variation will require greater thought. Hopefully through the assessment of the simplest cases, more clear drivers of differential rates of stutter can be obtained. Such assessments may need to take into account DNA secondary structure, or other nonproximal effects, to uncover the ultimate drivers of stutter. Lastly, analyzing stutter on different haplotypic backgrounds allows for a better understanding of stutter variance associated with particular alleles and may allow for better modeling for probabilistic genotyping.

## Figures and Tables

**Table 1:** Loci with alleles that contain a single repeating subunit, their marginal counts, and the number of different flanking haplotypes observed in the populations considered in this analysis.

Locus	Number of Alleles Considered (Total)	Number of Unique Flanking Haplotypes
D3S1358	40	1
D17S1301	183	1
CSF1PO	326	1
TH01	374	1
D10S1248	386	1
TPOX	566	1
DXS8378	127	2
HPRTB	216	2
D4S2408	307	2
D5S818	362	2
DXS7132	219	3
D9S1122	229	3
D20S482	249	3
D19S433	372	3
D6S1043	447	3
D1S1656	562	3
D2S441	310	4
DYS385	321	4
D8S1179	421	4
PENTA D	664	4
PENTA E	953	4
D16S539	424	5
D7S820	445	5
D18S51	798	5
D13S317	510	6
DXS10074	184	7

**Table 2:** Characterization of common flanking haplotypes.

Type of Change	Location of Change	Locus	Repeat Motif	Plausible Evolutionary Explanation
CT Deletion	Nonadjacent	D19S433	CCTT	Indel in flank
A → C	Nonadjacent	D16S539	GATA	SNP in flank
G → A	Nonadjacent	D7S820	TATC	SNP in flank
AAAGAAAAAAAAG Deletion	Adjacent	PENTAD	AAAGA	Indel (non-slippage)
TCTGTCTA Insertion	Adjacent	D2S441	TCTA	SNP in repeat
TCTATCTG Insertion	Adjacent	D8S1179	TCTA	SNP in repeat
TAGATCGA Deletion	Adjacent	D9S1122	TAGA	SNP in repeat
AATC Deletion	Adjacent	D13S317	TATC	SNP in repeat or indel
CCTA Deletion	Adjacent	D1S1656	TCTA	SNP in repeat or indel
CTCT Deletion	Adjacent	D5S818	ATCT	SNP in repeat or indel

The two most frequent flanking haplotypes found within a locus were compared, and the type of change required to transform the most common haplotype (index 1) into the second-most common haplotype (index 2) is shown (given a constant longest uninterrupted stretch (LUS)). The location of the change could also be adjacent to the repeat region, or nonadjacent, with ambiguities between the two being classified as adjacent. Full flanking haplotypes are shown in the supplementary materials. Note that the type of change depicted likely has little evolutionary bearing, and is instead meant to describe a difference between two haplotypes within a locus that have the same LUS. A plausible evolutionary mechanism is also shown so as to explore the origins of these differences, though a lack of knowledge on the ancestral (original) state and the exact mutational processes involved makes such explanations merely conjecture. SNP: single nucleotide polymorphism. Indel: Insertion/Deletion polymorphism.

**Table 3:** Results from robust linear regression modeling stutter as a function of the PAL.

Locus	Max VIF	R^2^	Intercept, Haplotype 1	Intercept, Haplotype 2	*p*-Value (Same Intercept)	PAL Slope, Haplotype 1	PAL Slope, Haplotype 2	*p*-Value (Same Slope)	Slope, Correct	*p*-Value (Correct)
D13S317	1.2	0.761	−0.031	−0.028	**5.7 × 10^−06^**	0.006	--	--	7.69 × 10^−07^	0.263
D16S539	1.2	0.794	−0.053	−0.051	0.173	0.015	--	--	−1.41 × 10^−06^	0.277
D19S433	1.2	0.735	−0.083	−0.083	0.995	0.013	--	--	−2.24 × 10^−06^	**0.025**
D1S1656	1.7	0.606	−0.063	−0.072	**0.005**	0.015	--	--	−1.38 × 10^−05^	0.154
D2S441	1.4	0.221	−0.035	−0.027	**6.6 × 10^−13^**	0.006	--	--	2.66 × 10^−06^	**0.008**
D5S818	1.6	0.445	−0.023	−0.047	**0.009**	0.007	0.009	0.008	3.80 × 10^−06^	0.466
D7S820	1.2	0.654	−0.040	−0.042	0.125	0.009	--	--	1.36 × 10^−07^	0.952
D8S1179	1.9	0.695	−0.045	−0.105	**1.2 × 10^−88^**	0.018	--	--	1.30 × 10^−06^	0.514
D9S1122	1.1	0.684	−0.078	−0.038	**5.5 × 10^−48^**	0.013	--	--	−4.06 × 10^−06^	0.287
PENTAD	2.3	0.481	−0.017	0.010	**2.0 × 10^−55^**	0.003	--	--	5.31 × 10^−07^	0.602

Two separate robust linear regressions were run on each locus: the first models *SR* ~ *Flank + PAL + C*, while the second models *SR* ~ *Flank × PAL + C* (see Appendix A). The first model allows each flanking haplotype’s intercepts to vary (the level of statistical support is seen in the first *p*-value) but they have the same slope, while the second model allows for different intercepts and slopes (the level of statistical support is seen in the second *p*-value) (see also Figure 1 and Figure 5). As the two models are nested, if the second model yielded significantly better fit, it was selected (D5S818), otherwise the first model was selected (remaining loci, estimates demarcated as --). The inferred model coefficients were translated into the above as per Appendix A. Variance inflation factors (VIFs), adjusted R^2^, as well as the (unscaled) coefficient estimates and their significance (bolded) are reported. As the coefficients are unscaled (in their original units), a point estimate of the effect of flanking variation relative to the effect of the PAL can be derived. For example, the two flanking haplotypes in the locus D9S1122 have difference in intercepts that is ~0.040. With respect to stutter, this difference roughly equals the effect of having an additional three repeat units (0.013 × 3).

**Table 4:** Results from robust linear regression modeling stutter as a function of the LUS.

Locus	Max VIF	R^2^	Intercept, Haplotype 1	Intercept, Haplotype 2	*p*-Value (Same Intercept)	LUS Slope, Haplotype 1	LUS Slope, Haplotype 2	*p*-Value (Same Slope)	Slope, Correct	*p*-Value (Correct)
D13S317	1.4	0.761	−0.031	−0.033	**0.024**	0.006	--	--	7.69 × 10^−07^	0.263
D16S539	1.2	0.794	−0.053	−0.051	0.173	0.015	--	--	−1.41 × 10^−06^	0.277
D19S433	1.2	0.735	−0.059	−0.069	**2.7 × 10^−08^**	0.012	--	--	−2.24 × 10^−06^	**0.025**
D1S1656	1.2	0.606	−0.049	−0.072	**6.4 × 10^−19^**	0.015	--	--	−1.38 × 10^−05^	0.154
D2S441	6.4	0.221	−0.035	−0.016	**4.6 × 10^−17^**	0.006	--	--	2.66 × 10^−06^	**0.008**
D5S818	1.4	0.445	−0.023	−0.056	**0.001**	0.007	0.009	0.008	3.80 × 10^−06^	0.466
D7S820	1.2	0.654	−0.040	−0.042	0.125	0.009	--	--	1.36 × 10^−07^	0.952
D8S1179	1.1	0.695	−0.045	−0.068	**1.2 × 10^−35^**	0.018	--	--	1.30 × 10^−06^	0.514
D9S1122	1.3	0.684	−0.053	−0.038	**2.4 × 10^−10^**	0.013	--	--	−4.06 × 10^−06^	0.287
PENTAD	1.5	0.481	−0.017	0.001	**5.7 × 10^−41^**	0.003	--	--	5.31 × 10^−07^	0.602

Two separate robust linear regressions were run on each locus: the first models *SR* ~ *Flank + LUS + C*, while the second models *SR* ~ *Flank × LUS + C* (see Appendix A). The first model allows each flanking haplotype’s intercepts to vary (the level of statistical support is seen in the first *p*-value) but they have the same slope, while the second model allows for different intercepts and slopes (the level of statistical support is seen in the second *p*-value) (see also Figure 1 and Figure 5). As the two models are nested, if the second model yielded significantly better fit, it was selected (D5S818), otherwise the first model was selected (remaining loci, estimates demarcated as --). The inferred model coefficients were translated into the above as per Appendix A. Variance inflation factors (VIFs), adjusted R^2^, as well as the (unscaled) coefficient estimates and their significance (bolded) are reported.

## Supplementary Materials

The following are available online at www.mdpi.com/2073-4425/8/11/329/s1. Table S1: Common flanking haplotypes (5′ and 3′) in commonly assayed forensic markers. The flanking index is given (1 or 2), as well as the type of change required to convert the first flanking haplotype to the second. The locus is given, as well as the count of the number of supporting haplotype calls.

## Appendix A

Within a locus, the stutter ratio can be modeled using linear regression as:

$$SR=b_{0}+b_{1}LUS+b_{2}Flank$$

where LUS is the length of the longest uninterrupted stretch (though the PAL could substitute the LUS and have the equivalent meaning), the *Flank* variable is an indicator variable (i.e., a dummy), that is 0 in the case that is inferred to the first flanking haplotype, and 1 in the case that is inferred to the second flanking haplotype. This type of model is a shared slopes model, and this name becomes apparent when considering the two cases:

Case 1: Flanking haplotype 1 (*Flank* is set to 0):

$$SR=b_{0}+b_{1}LUS+b_{2}0$$

$$SR=b_{0}+b_{1}LUS$$

Case 2: Flanking haplotype 2 (*Flank* is set to 1):

$$SR=b_{0}+b_{1}LUS+b_{2}1$$

$$SR=(b_{0}+b_{2})+b_{1}LUS$$

Thus, both flanking haplotypes are permitted the same slope (*b*_1_), but different intercepts. Whether or not these intercepts are significantly different lies in the significance of the *b*_2_ term.

Different flanking haplotypes can also be permitted to have both different slopes and different intercepts by incorporating an interaction term (*x*):

$$SR=b_{0}+b_{1}LUS\;x\;b_{2}Flank$$

This is equivalent to:

$$SR=b_{0}+b_{1}LUS+b_{2}Flank+b_{3}LUS*Flank$$

After collecting like terms, our two cases can now be expressed as two lines:

Case 1: Flanking haplotype 1 (*Flank* is set to 0):

$$SR=b_{0}+b_{1}LUS+b_{2}0+b_{3}LUS0$$

$$SR=b_{0}+b_{1}LUS$$

Case 2: Flanking haplotype 2 (*Flank* is set to 1):

$$SR=b_{0}+b_{1}LUS+b_{2}1+b_{3}LUS1$$

$$SR=(b_{0}+b_{2})+(b_{1}+b_{3})LUS$$

Thus, each flanking haplotypes is permitted a different intercept (*b*_0_ vs. *b*_0_ + *b*_2_) and different slope (*b*_1_ vs. *b*_1_ + *b*_3_), with significance of the variables *b*_2_ and *b*_3_ indicating whether or not the intercepts and slopes significantly differ, respectively. Note that an equivalent formulation of running 10 linear regressions, as we have done, is to encode nine out of the ten loci with indicator variables and add in the appropriate interaction terms, though this approach is perhaps less intuitive.
